# [^18^F]FAPI-42 PET/CT in differentiated thyroid cancer: diagnostic performance, uptake values, and comparison with 2-[^18^F]FDG PET/CT

**DOI:** 10.1007/s00259-022-06067-2

**Published:** 2022-12-10

**Authors:** Xingyu Mu, Xiaoxue Huang, Zewen Jiang, Meng Li, Lulu Jia, Zhongyuan Lv, Wei Fu, Jingsong Mao

**Affiliations:** 1 Department of Nuclear Medicine, Affiliated Hospital, Guilin Medical University, Guilin, China; 2 Department of Vascular Intervention, Affiliated Hospital, Guilin Medical University, Guilin, China; 3 Xiamen Key Laboratory of Endocrine-Related Cancer Precision Medicine, Xiamen, 361101 China

**Keywords:** [^18^F]FAPI-42, 2-[^18^F]FDG, Differentiated thyroid cancer, PET/CT

## Abstract

**Purpose:**

This study aimed to assess the diagnostic performance of [^18^F]FAPI-42 PET/CT and compare it with that of 2-[^18^F]FDG PET/CT in patients with differentiated thyroid cancer (DTC) with biochemical elevations in Tg or anti-Tg antibodies.

**Methods:**

A total of 42 patients with DTC with biochemical elevations in Tg or anti-Tg antibodies underwent [^18^F]FAPI-42 PET/CT as part of this study; of which, 11 additionally underwent 2-[^18^F]FDG PET/CT within 7 days. Images were semi-quantitatively and visually interpreted, and the quantity, location, and uptake values of lesions were noted. The diagnostic capacity of [^18^F]FAPI-42 PET/CT and biomarkers affecting the uptake of [^18^F]FAPI-42 were evaluated. In addition, the diagnostic performance and uptake of [^18^F]FAPI-42 and 2-[^18^F]FDG were compared, and the correlation between lesion diameter and quantitative parameters was investigated.

**Results:**

A total of 161 lesions were detected in 27 (64%) patients on [^18^F]FAPI-42 PET/CT. FAPI-positive local recurrence showed the highest uptake intensity, followed by lymphatic, other site-associated (bone and pleura), and pulmonary lesions (mean SUV_max_, 4.7 versus 3.7 versus 3.0 versus 2.2, respectively; *P* < 0.0001). The levels of TSH, Tg, and Tg-Ab did not affect the uptake value of lesions (median SUV_max_: 2.4 versus 3.2, *P* = 0.56; 2.9 versus 2.4, *P* = 0.0935; 2.8 versus 2.6, *P* = 0.0525, respectively). A total of 90 positive lesions were detected in 7 patients using both modalities. All positive lesions showed statistically higher uptake of 2-[^18^F]FDG than that of [^18^F]FAPI-42 (SUV_max_, 2.6 versus 2.1; *P* = 0.026). However, the SUV_max_ of [^18^F]FAPI-42 was higher than that of 2-[^18^F]FDG in local recurrences and lymphatic lesions (SUV_max_, 4.2 versus 2.9 and 3.9 versus 3.4, respectively; *P* > 0.05).

**Conclusion:**

[^18^F]FAPI-42 can be used for detecting lesions and reflecting FAP expression during local recurrence and metastasis in patients with DTC with biochemical elevations in Tg or anti-Tg antibodies. The diagnostic performance of [^18^F]FAPI-42 PET/CT is comparable with that of 2-[^18^F]FDG PET/CT in such patients.

**Supplementary Information:**

The online version contains supplementary material available at 10.1007/s00259-022-06067-2.

## Introduction

In 2020, approximately 586,000 thyroid cancer cases were reported worldwide, ranking ninth in terms of incidence [1]. Differentiated thyroid cancer (DTC) is the most common subtype, accounting for 80–85% of thyroid cancer cases, and its incidence has expended throughout recent many years [1, 2]. In spite of a generally decent prognosis, up to 30% of patients with DTC develop persistence or recurrence and 5–10% have the progressive, treatment-refractory disease [3]. Patients with DTC with suppressed thyroglobulin (Tg) levels of ≥ 1 ng/mL, stimulated Tg levels of ≥ 10 ng/mL, or increasing Tg-Ab levels are considered to have a biochemical incomplete response after total thyroidectomy and radioiodine remnant ablation. Approximately 20% of these patients develop structural disease, which is related to a poor prognosis [4]. Therefore, accurate and facile strategies of imaging are required for visualising local recurrences and metastatic lesions in patients with abnormal Tg or rising anti-Tg antibody levels.

Cancer-associated fibroblasts (CAFs) are crucial for the growth and progression of several tumours [5, 6]. Previous studies have indicated that the expression of CAFs is profoundly connected with aggressive outcomes in DTC [7, 8]. According to the American Thyroid Association (ATA) guideline recommendations, 2-[^18^F]FDG PET/CT should be thought of as a recommendation in a patient with elevated Tg with negative radioactive iodine (RAI) imaging. However, this modality may not directly allow the visualisation of CAFs expression [4]. Fibroblast activation protein (FAP) is overexpressed on CAFs and rarely expressed in normal tissues. Radionuclide-labelled fibroblast activation protein inhibitor (FAPI) can be taken up by multiple types of cancers [6], including thyroid cancer. Moreover, a previous study reported promising results of FAPI-based targeted therapy in thyroid cancer [9]. However, the efficacy of [^68^Ga]Ga-FAPI PET/CT in detecting lesions and guiding radioligand therapy of thyroid cancer remains controversial. Some studies have suggested that low uptake values of [^68^Ga]Ga-FAPI or [^68^Ga]Ga-FAPI-negative lesions are observed in thyroid cancer [6, 10], whereas other studies have indicated that [^68^Ga]Ga-FAPI PET/CT is a promising tool for detecting metastatic thyroid cancer [9, 11–15].

Fluorine-18 (^18^F)-labelled ligands provide some significant advantages over the now widely used ^68^Ga-labeled ligands. These advantages include not only an increase in examination owing to increased production capacity but also outstanding image quality. The latter is the result of optimum tracer doses, resulting in elevated imaging statistics and ^18^F decay properties. The positron emission energy of ^18^F is 0.6 MeV. Therefore, the distance required for positron deceleration in human tissues is significantly less than that required for ^68^Ga (*β* + energy = 2.3 MeV), which improves image resolution [16]. Furthermore, cyclotron utilisation is becoming more popular in China, and the fluorine standard has a high employment rate, which helps to promote the widespread use of such tracers [17]. Recently, several ^18^F-labelled tracers targeting FAPI have been described for clinical application in various cancers [17]. However, the diagnostic performance of [^18^F]FAPI PET/CT in DTC remains unclear. Therefore, this gap impels further investigation into the clinical meaning of [^18^F]FAPI PET/CT in DTC and determines which part of DTC is more sensitive to [^18^F]FAPI PET/CT than other imaging modalities.

The first aim of this study is to investigate the detection performance [^18^F]FAPI-42 PET/CT in patients with DTC with biochemical elevations in Tg or anti-Tg antibodies, and the second aim is to compare it with that of 2-[^18^F]FDG PET/CT in part of patients.

## Materials and methods

### Patients

This study was approved by the Institutional Review Board of the Affiliated Hospital of Guilin Medical University (institutional review board number: 2022WJWZCLL-01) and was approved by the institutional review board and registered on the Chinese Clinical Trial Registry website (http://www.chictr.org.cn, number ChiCTR2200063441). All patients signed an informed consent form prior to participating, and all procedures were carried out according to the Helsinki Declaration. Patients were consecutively recruited from October 2021 to May 2022 at the Affiliated Hospital of Guilin Medical University. The inclusion criteria were as follows: (i) patients with pathologically confirmed DTC who had received thyroidectomy (followed by ^131^I ablation); (ii) patients with high Tg levels (suppressed Tg levels ≥ 1 ng/mL or stimulated Tg levels ≥ 10 ng/mL) and increasing Tg-Ab levels (TgAb > 115 IU/mL considered positive), irrespective of TSH levels during the follow-up. The exclusion criteria were as follows: (i) patients without DTC; (ii) patients unwilling to undergo [^18^F]FAPI PET/CT; (iii) patients with secondary cancer; (iv) patients who did not provide written informed consent. Data, including demographic characteristics, tumour characteristics, and clinical information, were collected from medical records.

### Pharmaceutical synthesis

^18^F was produced in situ using a GE PET-trace 800 cyclotron system (GE, US) via irradiation of ^18^O-H2O with 16.5-MeV protons. The radiolabelling precursors of 2-[^18^F]FDG were provided by Shaanxi Zhengze Biotechnology Co., Ltd. 2-[^18^F]FDG was synthesised in our laboratory using X5-FDG (Shaanxi Zhengze Biotechnology Co. Ltd., China) module. The radiolabelling precursors of [^18^F]FAPI-42 were obtained from Jiangsu Huayi Technology Co. (Jiangsu, China) with high chemical purity (> 95%). A detailed description of the radiosynthesis process (GE modules) and the quality control of the [^18^F]FAPI-42 has been provided in prior studies [18, 19]. Radiochemical purity was determined using radio-TLC (AR-2000, BIOSCAN, USA) and radio-HPLC (UVIS-201, Alltech, USA), which was found to be ≥ 95%.

### PET/CT protocol

All 2-[^18^F] FDG PET/CT acquisitions were conducted in accordance with the international guidelines of the European Association of Nuclear Medicine [20]. Patients were not allowed to eat any food at least 6 h before the start of FDG PET/CT (i.e. with respect to the time of injection of FDG), thereby maintaining venous blood glucose levels of < 11 mmol/L (200 mg/dL) before 2-[^18^F]FDG administration. However, this was not required for [^18^F]FAPI-42 PET/CT acquisition. After injection of [^18^F]FAPI-42 with an activity of 215 MBq (range, 185–303 MBq) and 2-[^18^F]FDG with an activity of 246 MBq (range, 166–355 MBq), whole-body images were taken from the head to the middle of the thigh at approximately 1 h. All images were obtained using the Ingenuity TF PET/CT system (Philips, Amsterdam, Holland). After CT images were obtained (CT parameters: 120 kV, 250 mAs/slice, 600-mm transaxial FOV, no gap, collimation of 64 × 0.625 mm, the pitch of 0.8, rotation time of 0.75 s, slice thickness of 1 mm, and 512 × 512 matrices), PET images were taken at the bedside at 2.5 min in the same position to include the same regions (PET parameters: 3D FOV, 20 cm, ordered subset expectation-maximisation algorithm [OSEM], 3 iterations/12 subsets, full width at half maximum [FWHM], 3 mm). All patients were asked to drink water after the injection and urinate immediately before imaging.

### Imaging interpretation

The acquired CT and PET images were transferred to the MedEx system (MedEx Technology Limited Corporation, China) for registration, fusion, and measurement. All [^18^F]FAPI-42 and 2-[^18^F]FDG PET/CT examinations were independently reviewed by two certified nuclear medicine physicians with over 5 years of experience in nuclear oncology. Any difference in opinion was resolved by consensus. Image interpretation included visual and semiquantitative analyses. For the visualisation of lesions, increasing radioactivity compared with the background uptake (not explained by physiologic uptake) were considered positive lesions. The lesion uptake values were quantified based on the maximum standardised uptake values (SUV_max_) for both [^18^F]FAPI-42 and 2-[^18^F]FDG PET/CT scans, whereas those of normal organs were quantified based on SUV_mean_. SUV values were determined by drawing volumes of interest (VOIs) on metastatic lesions observed on [^18^F]FAPI-42 and 2-[^18^F]FDG PET/CT scans. Circular regions of interest (ROIs) were placed on axial slices around lesions with avid-lesion and were automatically integrated into a 3D VOI (MedEX system). Tumour-to-background ratios (TBRs) were determined to quantify the image contrast and were calculated for local recurrences (relative to the cervical muscle), metastasis in lymph nodes (relative to the cervical muscle), bones (relative to the L5 vertebra spongiosa), and the lungs (relative to the lung parenchyma).

### Statistical analysis

All statistical analyses were performed using the SPSS Statistics (version 25; IBM, Armonk, NY, USA), Excel for Windows (version 15.41; Microsoft, Redmond, Washington, USA), and GraphPad Prism 9.0 (GraphPad Software Corporation) software. The uptake of [^18^F]FAPI-42 and 2-[^18^F]FDG by normal tissues and positive lesions and TBRs were compared using the Wilcoxon signed-rank test. The uptake values of lesions with different BRAF_V600E_ mutations were compared using the Mann–Whitney *U* test. The correlation between the lesion size and SUV_max_ was analysed via Spearman’s correlation analysis. Two-tailed *P*-values of < 0.05 were considered statistically significant.

## Results

### Patient characteristics

**Fig. 1:**
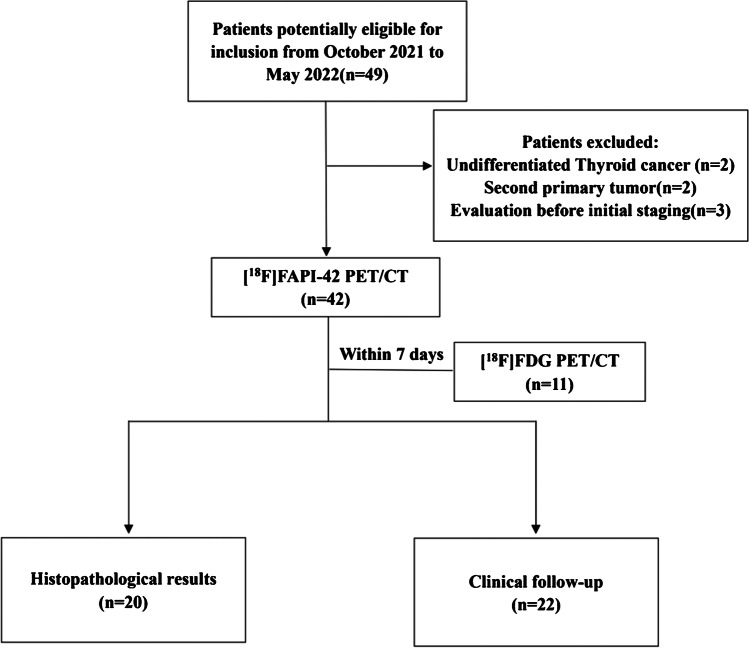
Flow chart for participant selection

A total of 42 patients (median age, 45 years; range, 12–75 years; 16 [38%] men and 26 [62%] women) underwent [^18^F]FAPI-42 PET/CT (Fig. 1); of which, 11 also underwent 2-[^18^F]FDG PET/CT within 7 days, and no patient received treatment during this period. All participants underwent neck US and chest CT as per the standard imaging procedure, and their serum Tg and Tg-Ab levels were evaluated during the follow-up period after undergoing [^18^F]FAPI-42 PET/CT (range, 2–9 months). Detailed information is presented in Table 1.

**Table 1:** Patient characteristics

Characteristic	Value
No. of participants	42
Age (years)	
Median (range)	46 (12–75)
Sex	
M	16 (38%)
F	26 (62%)
Histopathologic findings	
Papillary thyroid carcinoma	36 (86%)
Follicular thyroid carcinoma	5 (12%)
Hürthle	1 (2%)
BRAF_v600E_	
Mutation	8 (19%)
Wild type	8 (19%)
Not detected	27 (62%)
TSH level	
0–1 μIU/mL	23 (54%)
> 1 μIU/mL	19 (46%)
Tg level	
0–10 ng/mL	24 (57%)
>10 ng/mL	18 (43%)
Tg-Ab level	
Positive	9 (21%)
Negative	33 (79%)
Initial risk stratification	
Low	4 (9%)
Intermediate	16 (38%)
High	22 (53%)
RAI treatment before PET (cycles)*	2 (1–6)
Cumulative dose of ^131^I (GBq) *	10.3 (3.7–25.9)
Patients’ status (before PET)	
BIR	29 (69%)
SIR	13 (31%)
Method of confirmation	
Histopathologic results	20 (48%)
Follow-up results	22 (52%)

Data are numbers with percentages in parentheses; *TSH*, thyroid stimulating hormone; *Tg*, thyroglobulin; *Tg-Ab*, antithyroglobulin antibodies; *RAI*, radioactive iodine; *BIR*, biochemical incomplete responses; *SIR*, structural incomplete responses. *Data are medians, with ranges in parentheses

### Adverse events

All patients tolerated both examinations fairly well, without any drug-related pharmacological effects or physiological responses and related symptoms during injection through the end of the examination.

### Detection rate and semiquantitative analysis of [^18^F]FAPI-42 PET/CT

**Fig. 2:**
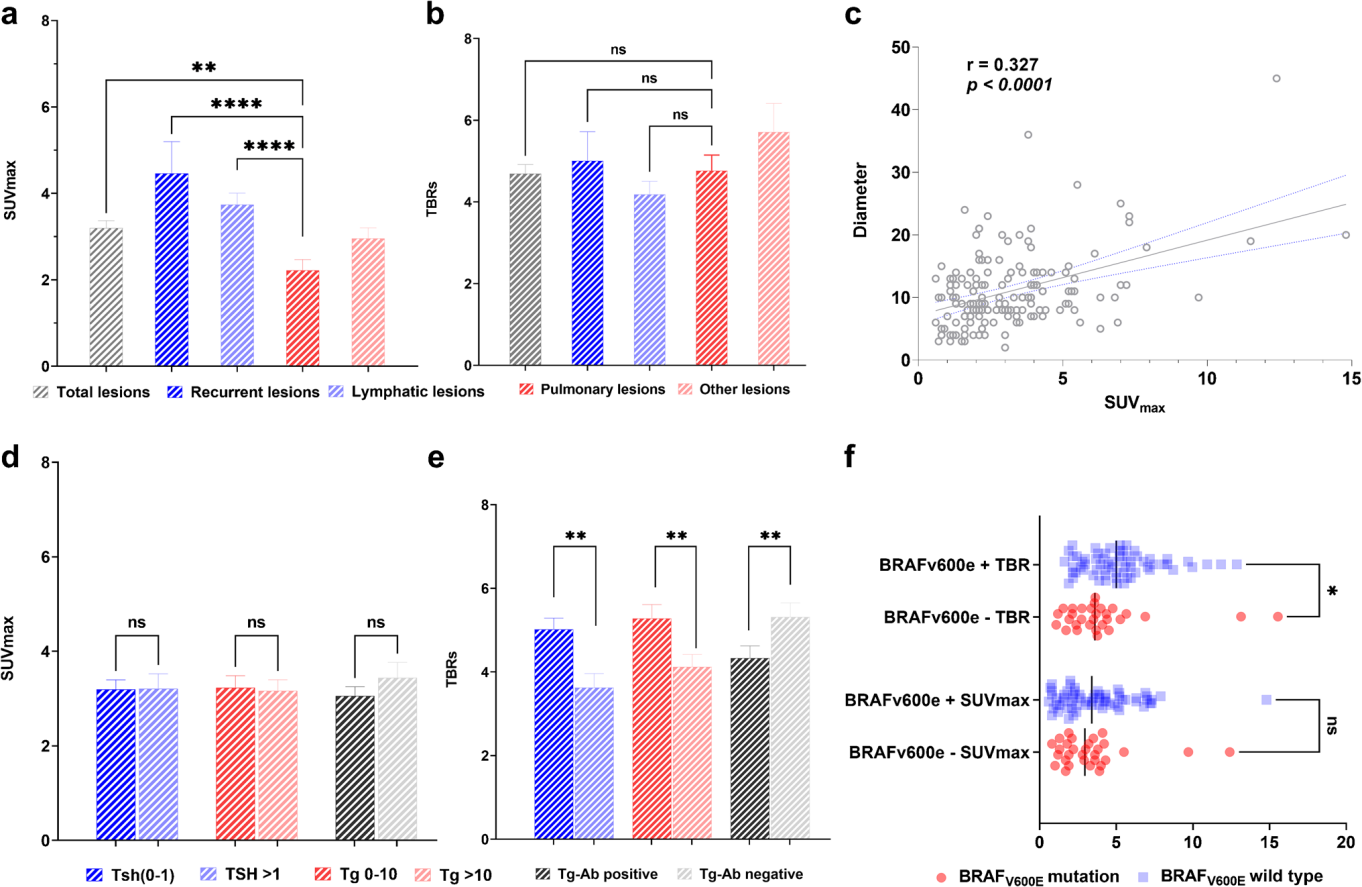
**a**, **b** Comparison of FAPI uptake among lesions localised in different sites. **c** Correlation between SUV_max_ and the diameter of positive lesions. **d**, **e** SUV_max_ and TBRs of all lesions based on the comparison of different clinical markers. **f** Differences in the SUV_max_ and TBRs of lesions between patients with BRAF_V600E_ mutation and wild-type BRAF_V600E_

A total of 161 positive lesions were detected in 27 (64%) patients on [^18^F]FAPI-42 PET/CT; of which, 16 (9.9%) were local recurrences at the primary site after thyroidectomy (followed by ^131^I ablation), 68 (42.3%) were lymphatic lesions localised in the neck (40/68, 58.8%) and chest (28/68, 41.2%), 52 (32.2%) were pulmonary lesions, and 25 (15.6%) were localised in other sites (bones and pleura). Overall, the mean SUV_max_ of positive lesions was 3.2, with a TBR of 4.7. The highest uptake intensity was seen in FAPI-positive local recurrences, followed by lymphatic lesions, lesions localised in other sites (bones and pleura), and pulmonary lesions (mean SUV_max_: 4.7 versus 3.7 versus 3.0 versus 2.2, respectively; *P* < 0.0001). Owing to the low background activity, the highest TBR was observed in lesions localised in bones and pleura, followed by local lesions, pulmonary lesions, and lymphatic lesions (mean TBR: 5.7 versus 5.0 versus 4.8 versus 4.18, respectively); however, no significant difference was observed (Fig. 2a). Additionally, the SUV_max_ of positive lesions showed a moderately positive correlation with lesion diameter (*r* = 0.327, *P* < 0.0001) (Fig. 2b).

A total of 38 and 123 positive lesions were detected in patients with elevated TSH levels (eTSH) and those with suppressed TSH levels (sTSH), respectively, on [^18^F]FAPI-42 PET/CT. The SUV_max_ of positive lesions was lower among patients with eTSH than among patients with sTSH (median SUV_max_, 2.4 versus 3.2; *P* = 0.56). However, a significant difference was observed in TBRs between patients with eTSH and sTSH (median TBRs, 3.6 versus 4.2; *P* = 0.007; Fig. 2e). A total of 79 and 82 lesions were detected in patients with low Tg levels (1–10 ng/mL) and those with high Tg levels (10–500 ng/mL), respectively, on [^18^F]FAPI-42 PET/CT. No significant difference was observed in FAPI uptake between patients with low and high Tg levels (median SUV_max_, 2.9 versus 2.4; *P* = 0.0935); however, TBRs were significantly different between the two groups of patients (median TBR, 5.0 versus 3.6; *P* = 0.002). Moreover, TBRs were higher among patients with positive Tg-Ab than among patients with negative Tg-Ab (median TBR, 5.2 versus 3.6; *P* = 0.003); however, no significant difference was observed in SUV_max_ between the two groups (median SUV_max_, 2.8 versus 2.6; *P* = 0.525; Fig. 2e). The activity of background between high and low Tsh level was represented in Supplementary Fig. 1. Furthermore, 16 patients who underwent [^18^F]FAPI-42 PET/CT had BRAF_V600E_ mutation. A total of 65 and 30 positive lesions were detected in patients with BRAF_V600E_ mutation and wild-type BRAF, respectively. The TBRs of both groups were different (median TBR, 5.0 versus 3.5; *P* = 0.012); however, SUV_max_ was not significantly different between the two groups (median SUV_max_, 3.0 versus 3.0; *P* = 0.952) (Fig. 2f).

### Comparison of the uptake of [^18^F]FAPI-42 and 2-[^18^F]FDG

**Fig. 3:**
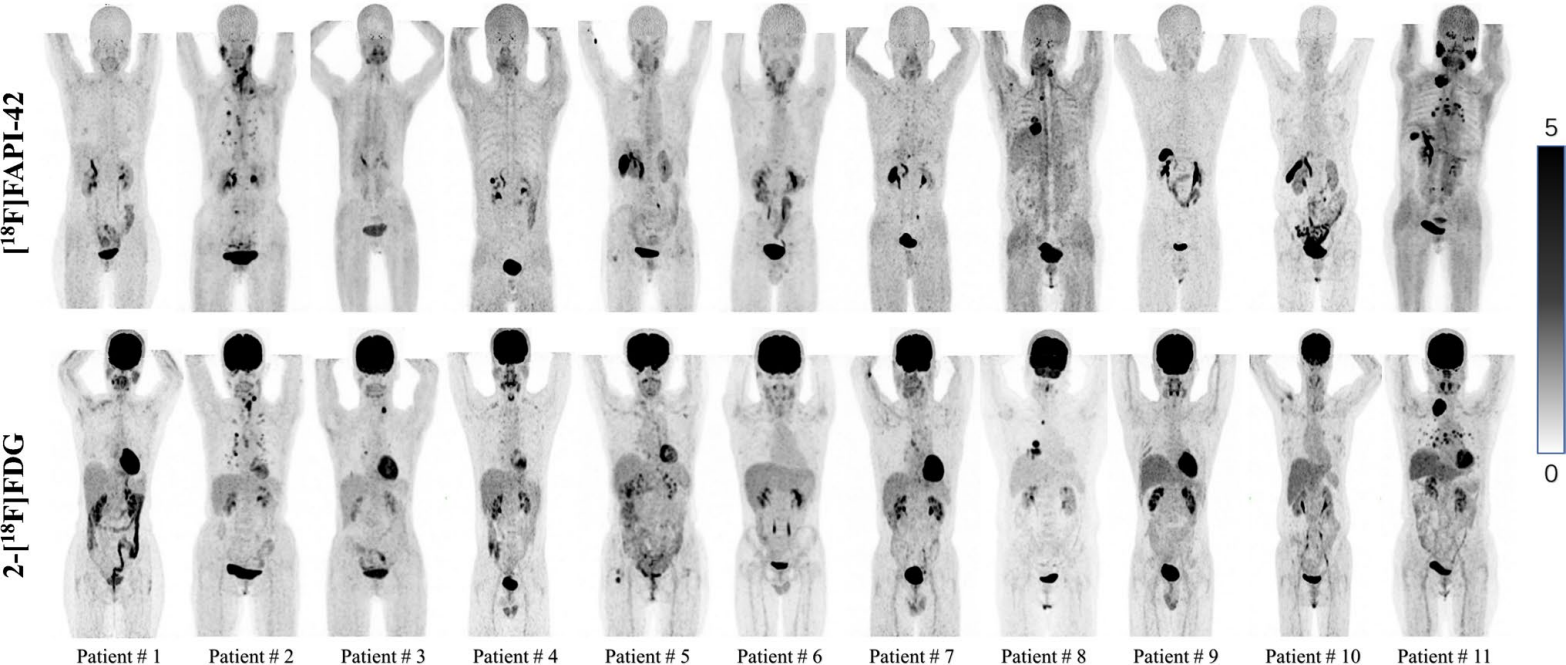
Maximum intensity projection images of all patients who underwent [^18^F]FAPI-42 and 2-[^18^F]FDG PET/CT

The maximum intensity projection images of all patients who underwent 2-[^18^F]FDG and [^18^F]FAPI-42 PET/CT are presented in Fig. 3. The biliary system is an important route for the excretion of [^18^F]FAPI-42, unlike 2-[^18^F]FDG. The brain, oral muscle, liver and L5 vertebra had lower uptake of [^18^F]FAPI-42 than that of 2-[^18^F]FDG (mean SUV_mean_, 0.1 versus 7.4; 1.4 versus 2.2; 0.9 versus 1.8; 0.8 versus 1.2, respectively; *P* < 0.05). The detailed parameters of normal tissues are presented in Supplementary Table 1 and Fig. 2a.

**Fig. 4:**
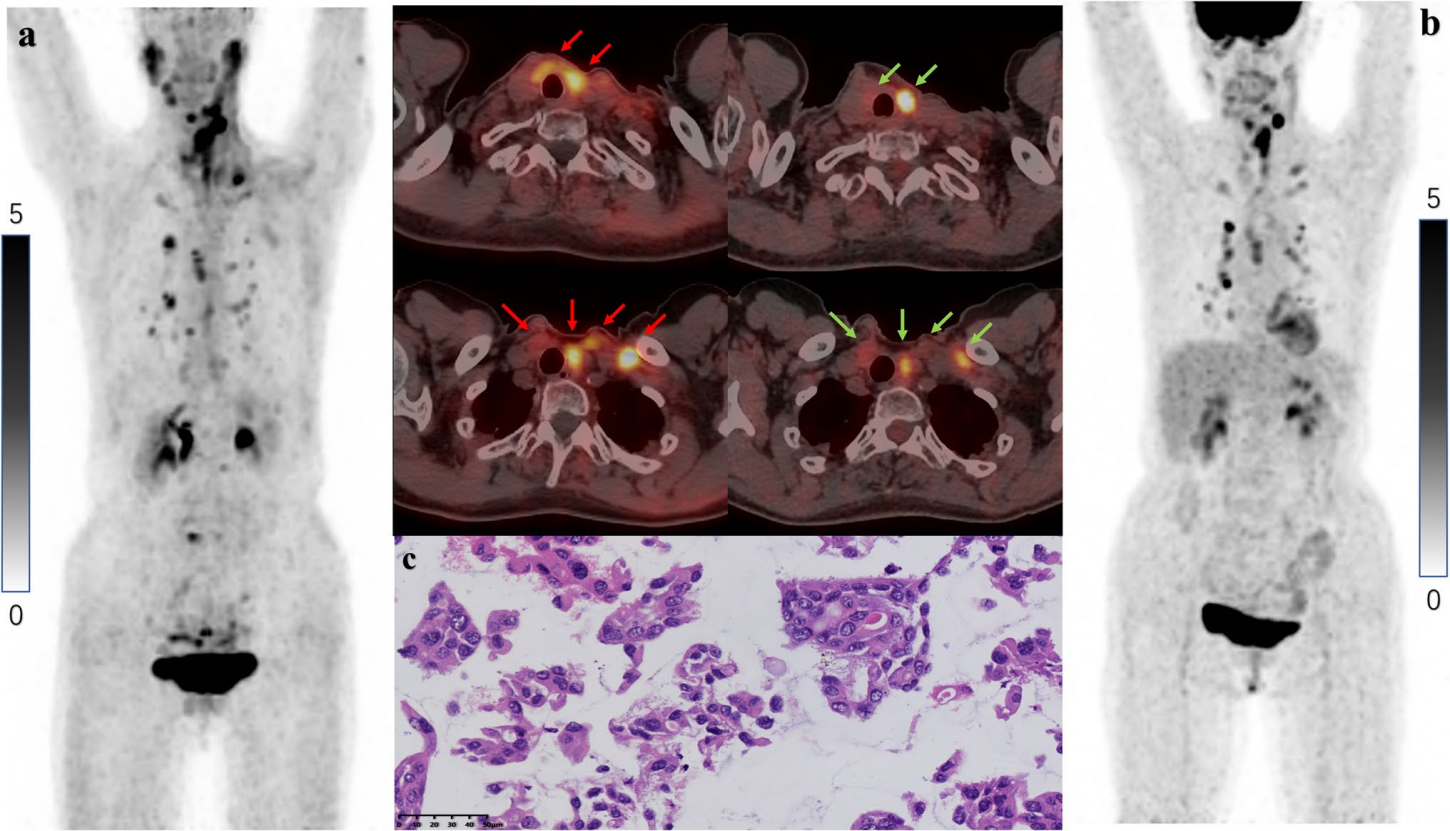
Local recurrences and metastatic lesions in a 66-year-old woman. **a** [^18^F]FAPI-42 PET/CT revealed local recurrence and metastatic lesions (in the lymph node and lung) with moderate-to-intense uptake (lesions are indicated by red arrows on axial fusion images). **b** 2-[^18^F]FDG PET/CT revealed fewer lesions and considerably lower radiotracer uptake than [^18^F]FAPI-42 PET/CT (lesions are indicated by green arrows on axial fusion images). **c** Pathologically confirmed diagnosis of PTC via US-FNA

**Fig. 5:**
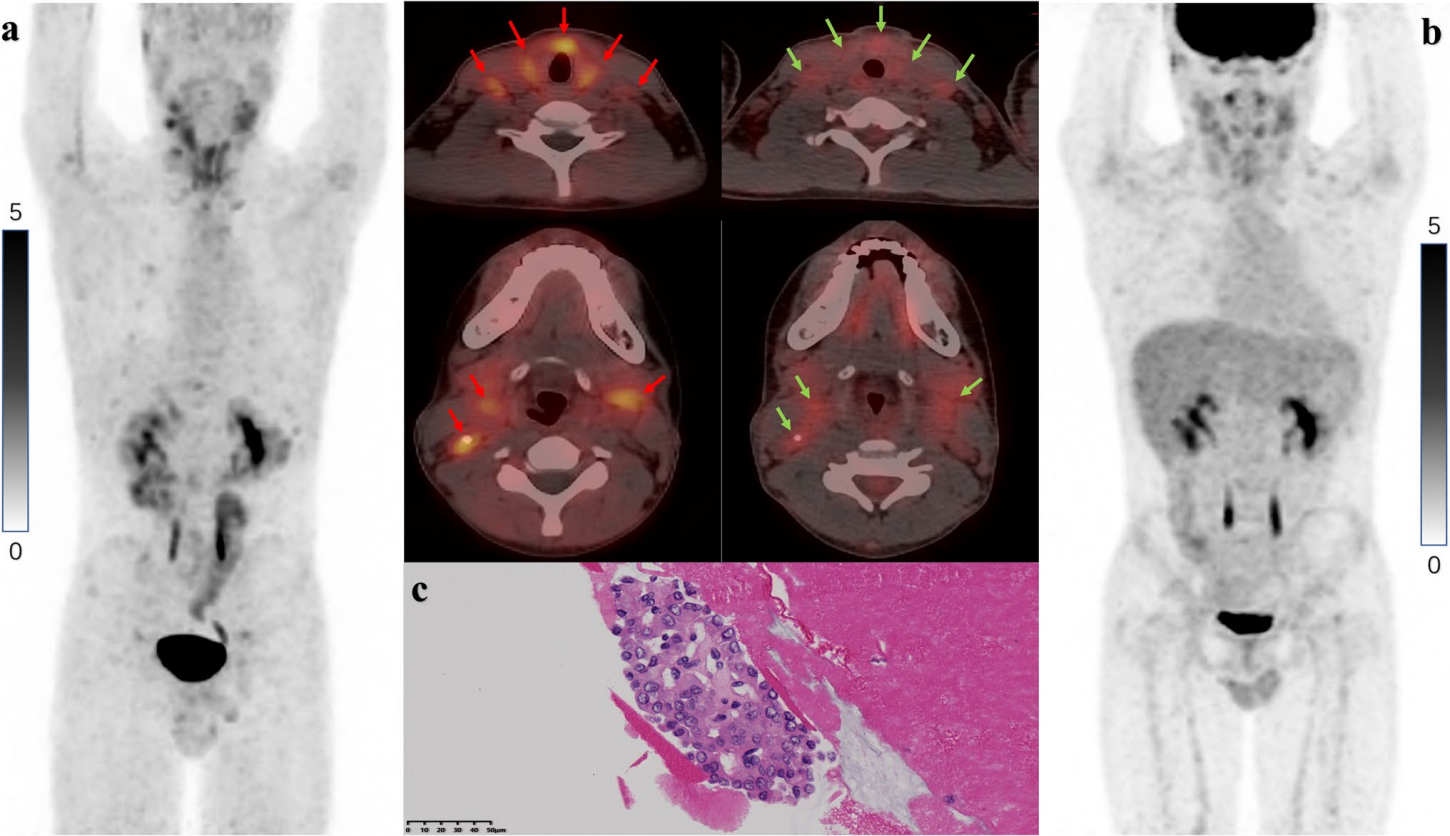
Local recurrences and lymphatic lesions in a 12-year-old boy (only this patient is younger than 18). **a** [^18^F]FAPI-42 PET/CT revealed local recurrence and lymphatic lesions with moderate-to-intense [^18^F]FAPI-42 uptake (lesions are indicated by red arrows on axial fusion images). **b** The uptake of 2-[^18^F]FDG was lower than that of [^18^F]FAPI-42 (lesions are indicated by green arrows on axial fusion images). **c** Pathologically confirmed diagnosis of PTC via US-FNA

**Fig. 6:**
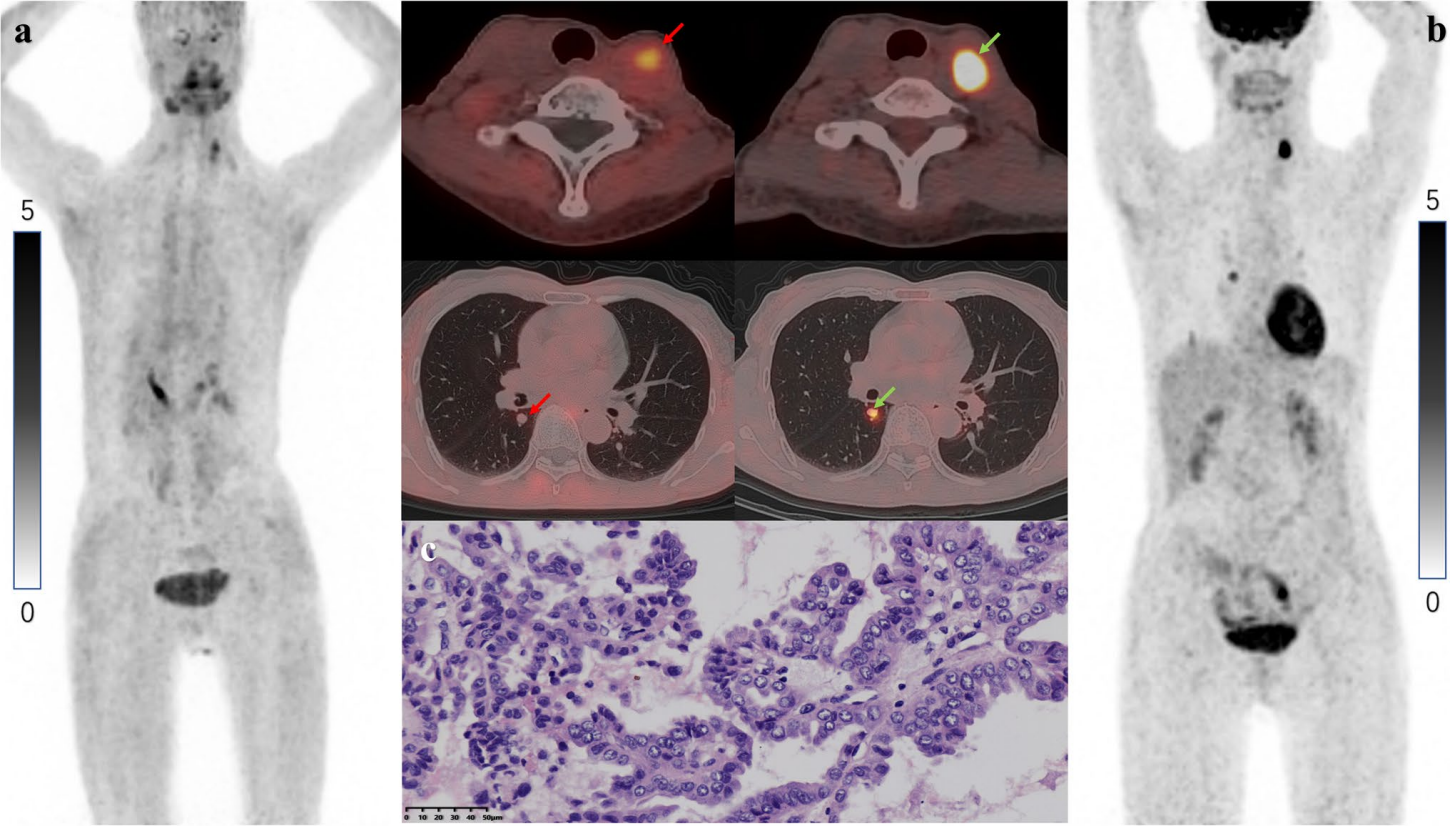
Metastatic lesions in a 67-year-old woman. **a** [^18^F]FAPI-42 PET/CT revealed local lymphatic and pulmonary lesions with low-to-moderate [^18^F]FAPI-42 uptake (lesions indicated by red arrows on axial fusion images). **b** The uptake of 2-[^18^F]FDG was higher than that of [^18^F]FAPI-42 (lesions are indicated by green arrows on axial fusion images). **c** Pathologically confirmed diagnosis of PTC via US-FNA

Table 2 shows a detailed comparison of SUV_max_ and TBRs among positive lesions. A total of 90 positive lesions were detected in 7 patients. Moreover, 2 lesions were not detected on 2-[^18^F]FDG PET/CT, and 5 were not detected on [^18^F]FAPI-42 PET/CT. The SUV_max_ of 30% (27/90) positive lesions were higher for [^18^F]FAPI-42 than for 2-[^18^F]FDG, that of 23% (21/90) lesions was equal, and that of 47% (42/90) lesions was lower for [^18^F]FAPI-42 than for 2-[^18^F]FDG. The SUV_max_ of positive lesions had a moderately positive correlation between the two tracers (*r* = 0.651; *P* < 0.001; Supplementary Fig. 2b). The SUV_max_ of positive lesions (SUV_max_, 2.1 versus 2.6, respectively; *P* = 0.026; Supplementary Fig. 2c) and pulmonary lesions (SUV_max_, 1.3 versus 2.2, respectively; *P* = 0.002) was significantly different between [^18^F]FAPI-42 PET/CT and 2-[^18^F]FDG PET/CT. The TBRs of positive lesions (TBR, 3.7 versus 3.8, respectively; *P* = 0.025) and pulmonary lesions (metastasis to the lung parenchyma) (TBR, 3.3 versus 4.7, respectively; *P* < 0.001; Supplementary Fig. 2d) were higher on 2-[^18^F]FDG PET/CT than on [^18^F]FAPI-42 PET/CT. Images representing higher uptake of [^18^F]FAPI-42 are shown in Figs. 4 and 5, whereas those representing higher uptake of 2-[^18^F]FDG are shown in Fig. 6. On the other hand, patients only underwent [^18^F]FAPI-42 PET/CT shown 76 positive lesions, and the distribution of positive lesions had a demonstration at Supplementary Table 2.

**Table 2:** Numbers and uptake values of lesions on [^18^F]FAPI-42 PET/CT and 2-[^18^F]FDG PET/CT

Lesion location and parameters	[^18^F]FAPI-42	2-[^18^F]FDG	*P*-value
Local recurrences			
No. of lesions	6	6	
Median SUV_max_	4.2 (2.5, 14.8)	2.9 (2.1, 27.4)	0.715
Median TBRs	4.4 (0.8, 14.8)	3.6 (1.0, 16.8)	0.6
Positive lymph nodes			
No. of lesions	37	39	
Median SUV_max_	3.9 (0.6, 10.7)	3.4 (0.8, 15.5)	0.652
Median TBRs	4.8 (0.8, 14.8)	4.5 (1.0,16.8)	0.34
Positive lung lesions			
No. of lesions	41	42	
Median SUV_max_	1.3 (0.4, 12.1)	2.2 (0.9, 17.3)	0.002**
Median TBRs	3.3 (1.2, 13.2)	4.7 (1.5, 34.5)	0.000***
Positive bone lesions			
No. of lesions	1	1	
SUV_max_	3.6	2.2	
Total positive lesions			
No. of lesions	85	88	
Median SUV_max_	2.1 (0.4, 12.1)	2.6 (0.6, 27.4)	0.026*
Median TBRs	3.7 (0.8, 14.8)	3.8 (1.0, 34.5)	0.025*

*, *P* < 0.05; **, *P* < 0.005; ***, *P* < 0.001

### ^131^I whole-body imaging

In our cohort, thirteen patients are treated by ^131^I treatment (dose range: 3.7–7.4 Gbq) after PET/CT. Compared with the post-therapeutic ^131^I whole-body scan, [^18^F]FAPI-42 PET/CT detected more positive lesions in 4 patients than the ^131^I whole-body scan. One patient was shown the same lesions, and five patients were not detected with positive lesions by dual imaging modalities. Unfortunately, three patients showed diffuse pulmonary uptake on post-therapeutic ^131^I whole-body scan, but the [^18^F]FAPI-42 PET/CT depicted false-negative results in the pulmonary disease of these patients (Supplementary Fig. 3). Among the three patients, one patient manifested positive metastatic lymph nodes by [^18^F]FAPI-42 PET/CT, but a post-therapeutic ^131^I whole-body scan indicated negative results in metastatic lymph nodes. Another two patients manifested similar results of positive lesions (except pulmonary lesions) by two modalities (Supplementary Table 3).

### The result of negative patients during follow-up

Fifteen patients in our cohort did not find positive lesions by two imaging modalities. Among these patients, three patients converted to excellent response (ER) according to the 2015 ATA guidelines. Two patients were defined as having an indeterminate response (IR). Ten patients still maintain the situation of biochemical incomplete responses (BIR) until the latest follow-up. All patients have not found structural lesions by CT or US during follow-up (Supplementary Table 4). Representative imaging was presented in Supplementary Fig. 4.

## Discussion

This study demonstrated that [^18^F]FAPI-42 PET/CT can be used for detecting lesions and reflecting FAP expression of lesions in patients with DTC with biochemical elevations in Tg or anti-Tg antibodies. In particular, local, lymphatic, bony, and pleural lesions showed moderate-to-high uptake on [^18^F]FAPI-42 PET/CT. Owing to the low background activity, the TBR of pulmonary lesions was similar to that of lesions localised in other sites. TSH, Tg, and Tg-Ab levels did not affect the uptake value of lesions. Furthermore, patients with BRAF_V600E_ mutation had higher uptake of [^18^F]FAPI-42 than that of 2-[^18^F]FDG PET/CT, and the diagnostic performance of [^18^F]FAPI-42 PET/CT was comparable to that of 2-[^18^F]FDG PET/CT. In particular, SUV_max_ of local recurrences and lymphatic lesions was higher on [^18^F]FAPI-42 PET/CT than on 2-[^18^F]FDG PET/CT.

Radionuclide-labelled FAPI can be used for detecting FAP and CAFs, which are abundant in the tumour stroma of > 90% epithelial carcinomas [5, 6]. In this study, [^18^F]FAPI-42 PET/CT had a promising detection ability for lesions in patients with DTC with biochemical elevations in Tg or anti-Tg antibodies. This finding is consistent with that of previous studies investigating the diagnostic performance of [^68^Ga]Ga-FAPI PET/CT in thyroid cancer [11, 15]. In addition to an excellent rate of detection, other advantages of FAPI radioligands based on ^18^F include long half-life, greater imaging quality, economic convenience, greater availability, and consequent higher number of PET/CT examinations performed, which are promising alternatives and more practical to FAPI radioligands based on ^68^Ga in clinical practice [21, 22]. Furthermore, with the development of theranostics based on FAPI [13], [^18^F]FAPI-42 PET/CT provides an opportunity for diagnostic imaging to evaluate the therapeutic feasibility and benefits of FAPI-based targeted therapy.

Chen et al. [15] reported that the uptake of [^68^Ga]Ga-FAPI may be associated with Tg levels, which cause a low tumour burden. However, Fu et al. reported that FAP expression may not be associated with Tg levels. Consistently, in this study, FAPI uptake was not significantly different among patients with different Tg levels. Similarly, TSH and Tg-Ab levels did not affect the uptake of FAPI [23]. This difference can be explained by the fact that fibroblasts are genetically more stable and less resistant to therapeutic intervention than tumour cells [24]. Therefore, the uptake of [^18^F]FAPI-42 in tumorous lesions is not easily influenced by clinical factors and may be more stable than radioligands targeting tumour cells. Although the SUV_max_ of [^18^F]FAPI-42 in positive lesions was not affected by the level of biomarkers, the TBR of [^18^F]FAPI-42 manifested significantly a difference between different levels of biomarkers. Hence, we infer that the expression of FAP may be regulated by some biomarkers, such as TSH or thyroid hormone. Further research should be done to confirm the existence of an association between FAP and clinical factors.

In this study, the diagnostic performance of [^18^F]FAPI-42 PET/CT was comparable with that of 2-[^18^F]FDG PET/CT. As mentioned in a previous study, FAP expression is low in normal tissues, including the brain, oral mucosa, liver, and bones [21]. In this study, low background activity was observed in these organs. Therefore, the TBRs of lesions localised in these sites were higher. Chen et al. [11] reported that SUV_max_ of [^68^Ga]Ga-FAPI was higher than that of 2-[^18^F]FDG in most thyroid cancer lesions. However, in this study, the SUV_max_ and TBRs of malignant lesions were similar on [^18^F]FAPI-42 PET/CT and 2-[^18^F]FDG PET/CT. Furthermore, only 2 lesions were detected by [^18^F]FAPI-42 PET/CT, but not by 2-[^18^F]FDG PET/CT also different from that study, which showed [^68^Ga]FAPI PET/CT depicted a greater number of metastatic lesions than 2-[^18^F]FDG PET/CT. The main reason results in these discrepancies may be associated with the patient cohort. Compared with our study, the previous study had more patients with Tg elevation and negative iodine scintigraphy (TENIS) in their cohort. And, patients with higher Tg levels observed a higher detection rate of [^68^Ga]FAPI PET/CT than that of 2-[^18^F]FDG PET/CT. However, patients with a relatively lower Tg level may cause a comparable detection rate between the two modalities. Other reasons that lead to these discrepancies may be attributed to the differences in radionuclides and radioligands.

Some false-negative uptake values of pulmonary lesions were observed on [^18^F]FAPI-42 PET/CT; however, ^131^I whole body scan showed diffuse pulmonary uptake. Previous reports described a similar performance in ^124^I PET/CT [25, 26], which suggests the activity of pulmonary lesions was under the threshold of detectability/visibility. Also, we speculated that this phenomenon was associated with lesion diameter because these lesions had not properly formed a tumour stroma. Wu et al. [27] illustrated that dual-time-point ^124^I PET/CT imaging can improve the lesion detection rate in metastatic, differentiated thyroid cancer. Therefore, further studies may be needed to determine the imaging performance on [^18^F]FAPI-42 PET/CT at a different time point in pulmonary metastasis of DTC.

An earlier study reported that strong CAF-related protein expression is associated with the BRAF_V600E_ mutation in both PTC and CAF cells [28]. CAFs is activated in the conventional mutant BRAF_V600E_ DTC and influences carcinogenesis by modifying the extracellular matrix (ECM), improving growth factors, and secreting protease [7, 29]. A similar phenomenon was observed in this study: the uptake of [^18^F]FAPI-42 was higher among patients with BRAF_V600E_ mutation than among patients with wild-type BRAF_V600E_. Therefore, the uptake of [^18^F]FAPI-42 may be an effective parameter for predicting the mutation status of BRAF_V600E_ or detecting metastatic lesions in patients with BRAF_V600E_ mutation.

A previous study showed that FDG uptake might also be increased by CAFs in tumours [30]. Consistently, in this study, the SUV_max_ of lesions on [^18^F]FAPI-42 PET/CT was positively correlated with that on 2-[^18^F]FDG PET/CT. Shangguan et al. [30] reported that CAFs were found in the surgical margin of radical resection (5 cm away from the tumour lesion) in patients with colon cancer, which is often accompanied by high recurrence rates. Similarly, extra FAPI-avid lesions were observed surrounding FDG-avid lesions in patients with PTC. Therefore, FAPI-related radiotracers may have a better ability to predict recurrence as compared with 2-[^18^F]FDG in such patients. However, further studies are required to validate this phenomenon. Tumour size is associated with the uptake of 2-[^18^F]FDG [30, 31].

This study has several limitations. First, the number of participants included in this study was relatively small, especially the number of patients who underwent both modalities. Therefore, we collected lesions from different sites. Second, histopathological analyses did not allow the detection of all lesions. This is an inherent problem of clinical research because not every lesion can be safely biopsied or surgically removed [32]. Third, the evaluation for possible false-negative lesions was incomplete because non-invasive imaging was also taken as the reference standard for detecting lesions.

## Conclusion

^18^F-labelled FAPI is useful for detecting lesions and reflecting FAP expression of structural lesions in patients with DTC with biochemical elevations in Tg or anti-Tg antibodies. In addition, the performance of [^18^F]FAPI-42 PET/CT was comparable with that of 2-[^18^F]FDG PET/CT in depicting DTC lesions. To assess the therapeutic viability of FAPI-based targeted therapy, [^18^F]FAPI-42 PET/CT offers an option for diagnostic imaging of FAP.

## Supplementary Information

ESM 1 (DOCX 2008 kb)
