# Supplementary material for: [18F]FAPI-42 PET/CT in differentiated thyroid cancer: diagnostic performance, uptake values, and comparison with 2-[18F]FDG PET/CT
Source: Eur J Nucl Med Mol Imaging. 2022 Dec 10;50(4):1205–15. doi: 10.1007/s00259-022-06067-2 (PMC9931817; doi:10.1007/s00259-022-06067-2)
Supplement: Supplementary file 1 — (DOCX 2008 kb) [file 259_2022_6067_MOESM1_ESM.docx]

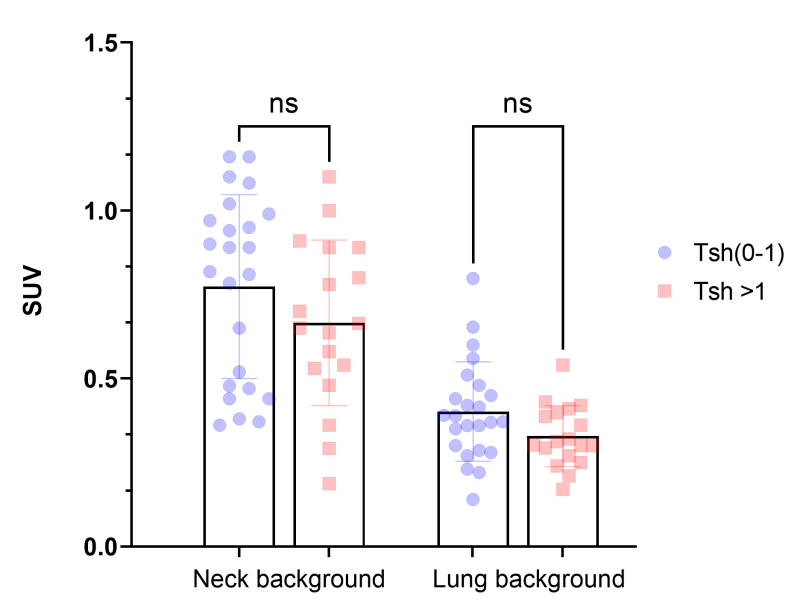


**Supplementary Fig. 1** The uptake of neck and lung background in different TSH levels on [^18^F]FAPI-42 PET/CT.


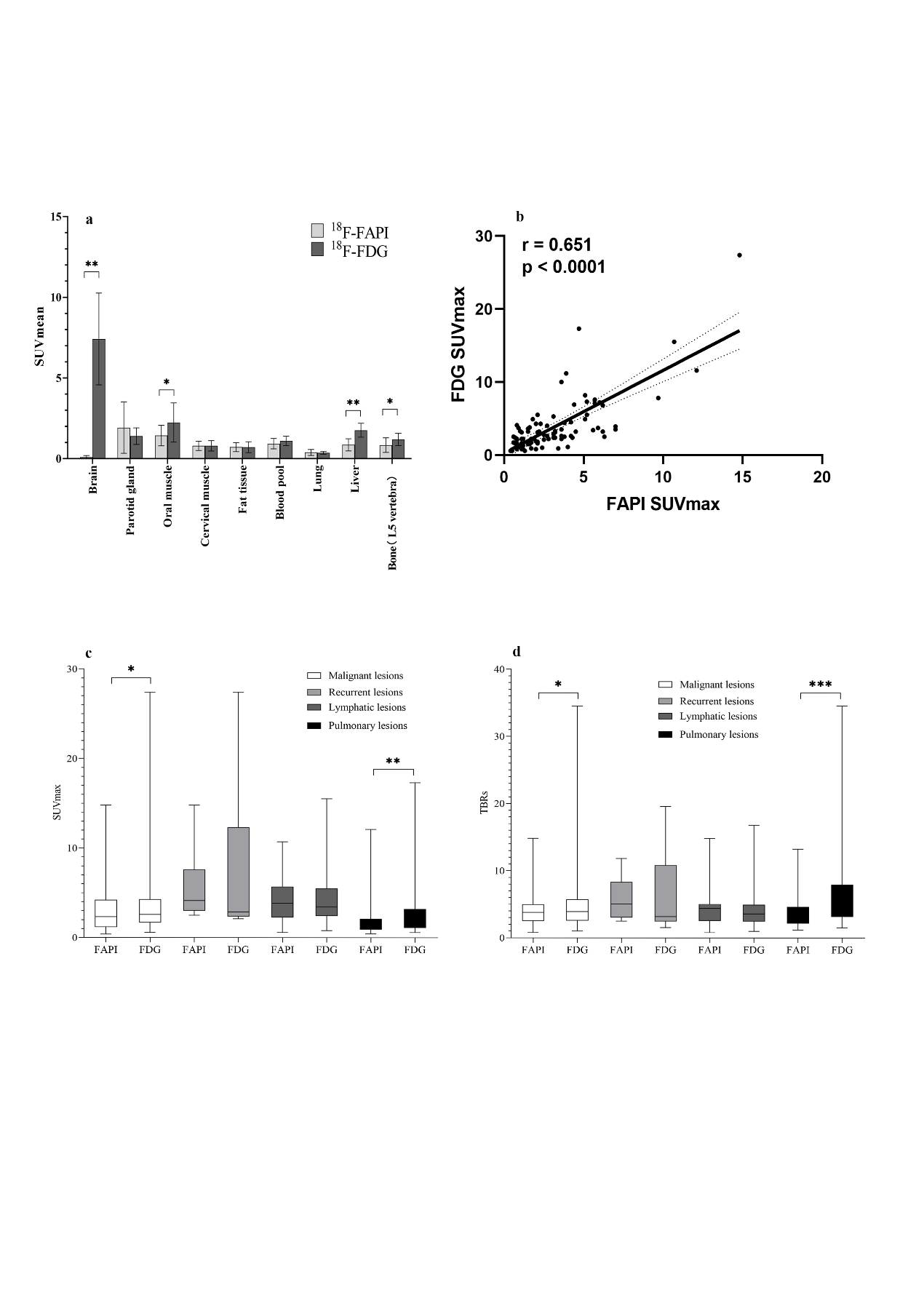


**Supplementary Fig. 2** (a) The uptake of [^18^F]FAPI-42 and ^18^F-FDG by different normal organs (*, P < 0.05; **, P < 0.005). All data are expressed as mean and standard deviation. (b) Comparison of SUV_max_ between [^18^F]FAPI-42 and 2-[^18^F]FDG. (c and d) The uptake of [^18^F]FAPI-42 and 2-[^18^F]FDG in different metastatic lesions (*, P < 0.05; **, P < 0.005; ***, P < 0.001).

| Tissue types | SUVmax (median) | | SUVmean (median) | | SUVmax (mean ± SD) | | | SUVmean (mean ± SD) | | |
| --- | --- | --- | --- | --- | --- | --- | --- | --- | --- | --- |
|  |  |  |  |  |  |  |  |  |  |  |
|  | [^18^F]FAPI-42 | 2-[^18^F]FDG | [^18^F]FAPI-42 | 2-[^18^F]FDG | [^18^F]FAPI-42 | 2-[^18^F]FDG | *p* value | [^18^F]FAPI-42 | 2-[^18^F]FDG | *p* value |
| Brain | 0.07 | 9.04 | 0.04 | 7.29 | 0.2 ± 0.2 | 9.68 ± 3.8 | 0.003 | 0.1 ± 0.1 | 7.7 ± 2.7 | 0.003 |
| Parotid gland | 1.63 | 1.54 | 1.24 | 1.51 | 1.9 ± 1.4 | 1.7 ± 0.6 | 0.859 | 1.6 ± 1.1 | 1.4 ± 0.5 | 0.859 |
| Oral muscle | 1.34 | 2.42 | 1.28 | 2.02 | 1.4 ± 0.4 | 2.2 ± 0.4 | 0.013 | 1.3 ± 0.4 | 2.0 ± 0.8 | 0.021 |
| Cervical muscle | 0.88 | 0.86 | 0.78 | 0.70 | 0.9 ± 0.3 | 1.0 ± 0.3 | 0.131 | 0.8 ± 0.2 | 0.7 ± 0.3 | 0.859 |
| Fat tissue | 0.87 | 0.68 | 0.79 | 0.65 | 0.7 ± 0.3 | 0.7 ± 0.3 | 0.929 | 0.7 ± 0.3 | 0.7 ± 0.3 | 0.929 |
| Blood pool | 1.07 | 1.43 | 0.84 | 1.14 | 1.0 ± 0.2 | 1.3 ± 0.3 | 0.075 | 0.9 ± 0.2 | 1.1 ± 0.3 | 0.110 |
| Lung | 0.53 | 0.57 | 0.35 | 0.39 | 0.6 ± 0.2 | 0.5 ± 0.1 | 0.594 | 0.4 ± 0.2 | 0.4 ± 0.1 | 0.894 |
| Liver | 1.17 | 2.22 | 0.87 | 1.56 | 1.2 ± 0.4 | 2.3 ± 0.4 | 0.004 | 0.8 ± 0.4 | 1.7 ± 0.3 | 0.004 |
| Bone (L5) | 1.15 | 1.45 | 0.86 | 1.28 | 1.1 ± 0.5 | 1.5 ± 0.4 | 0.050 | 0.8 ± 0.5 | 1.2 ± 0.4 | 0.026 |

**Supplementary Table 1.** The uptake of [^18^F]FAPI-42 and 2-[^18^F]FDG by healthy tissues


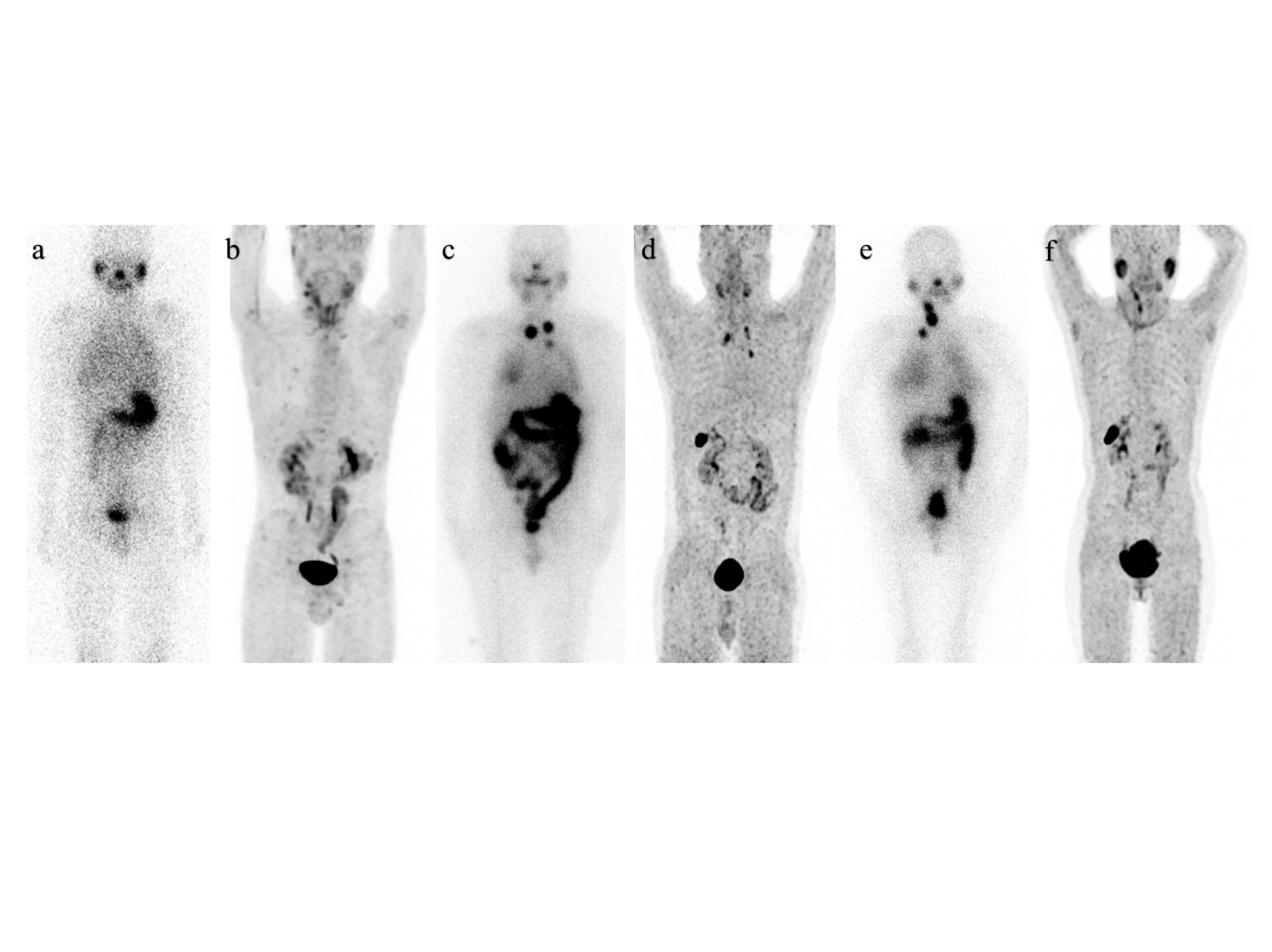
**Supplementary Fig. 3** (a ,b) ^131^I whole body scan and [^18^F]FAPI-42 PET/CT scan of a patient. ^131^I whole body scan shows diffuse pulmonary uptake, [^18^F]FAPI-42 PET/CT scan shows negative findings in the chest. A similar phenomenon was observed in other patients (c–f).

**Supplementary Table 2.** Number of lesions in positive patients underwent single [^18^F]FAPI-42 PET/CT

| Patient number | Local recurrence | Lymphatic lesions | Pulmonary lesions | Other lesions |
| --- | --- | --- | --- | --- |
| 1 |  |  |  | 4 |
| 2 |  | 3 |  |  |
| 3 |  | 3 |  |  |
| 4 |  |  |  | 8 |
| 5 | 1 |  |  |  |
| 6 | 1 | 2 |  |  |
| 7 |  |  | 2 | 6 |
| 8 |  | 1 |  |  |
| 9 |  | 2 |  |  |
| 10 |  | 1 |  |  |
| 11 | 2 |  |  |  |
| 12 | 1 |  |  |  |
| 13 |  | 3 | 5 |  |
| 14 | 1 | 2 |  |  |
| 15 |  | 5 | 1 |  |
| 16 |  | 2 |  | 2 |
| 17 |  |  | 3 | 4 |
| 18 | 1 |  |  |  |
| 19 | 1 | 3 |  |  |
| 20 | 2 | 4 |  |  |

**Supplementary Table 3.** [^18^F]FAPI-42 PET/CT vs. Post-therapeutic ^131^I whole-body scan

|  | | |
| --- | --- | --- |
|  | Patients number |  |
| FAPI superior | 4 |  |
| ^131^I superior | 3 |  |
| Equal | 6 |  |
| Total | 13 |  |
| *FAPI*: fibroblast activation protein | |  |

**Supplementary Table 4.** The follow-up result of patients without positive lesions by dual-radiotracers

| Patient number | Gender | Age | PET Tg（ng/ml） | PET Tg-Ab(IU/ml) | Follow-up Tg（ng/ml） | Follow-up Tg-Ab （ng/ml） | US/CT | Category |
| --- | --- | --- | --- | --- | --- | --- | --- | --- |
| 1 | F | 43 | 1.74 | 10.7 | 0.7 | 47.3 | NSE | IR |
| 2 | F | 33 | 11.8 | 51.4 | 12.5 | 64.6 | NSE | BIR |
| 3 | F | 63 | 2.26 | 13.9 | 1.16 | 12 | NSE | BIR |
| 4 | F | 33 | ＜0.04 | 1403 | ＜0.04 | 1413 | NSE | BIR |
| 5 | M | 32 | 1.27 | 25.1 | 4.3 | 10.5 | NSE | BIR |
| 6 | F | 68 | 1.46 | ＜10 | 0.76 | ＜10 | NSE | IR |
| 7 | M | 35 | 3 | ＜10 | 0.2 | 32.5 | NSE | ER |
| 8 | M | 29 | 8.89 | ＜10 | 8.4 | ＜10 | NSE | BIR |
| 9 | M | 40 | 8.19 | 11.9 | 8.9 | ＜10 | NSE | BIR |
| 10 | F | 61 | 14.5 | <10 | 19 | <10 | NSE | BIR |
| 11 | M | 46 | 2.14 | 11.7 | 0.09 | 20.1 | NSE | ER |
| 12 | F | 59 | <0.040 | 503 | <0.040 | 580 | NSE | BIR |
| 13 | M | 43 | 7.3 | 10.6 | 5.58 | <10 | NSE | BIR |
| 14 | F | 43 | <0.040 | 1316 | <0.040 | 1859 | NSE | BIR |
| 15 | F | 50 | 5.4 | 11.2 | 0.04 | <10 | NSE | ER |
| *Tg*: thyroglobulin, *Tg-Ab*: thyroglobulin anti-body, *NSE*: no structural evidence, *IR*: indeterminate response*, BIR*: biochemical incomplete response, *ER*: excellent response  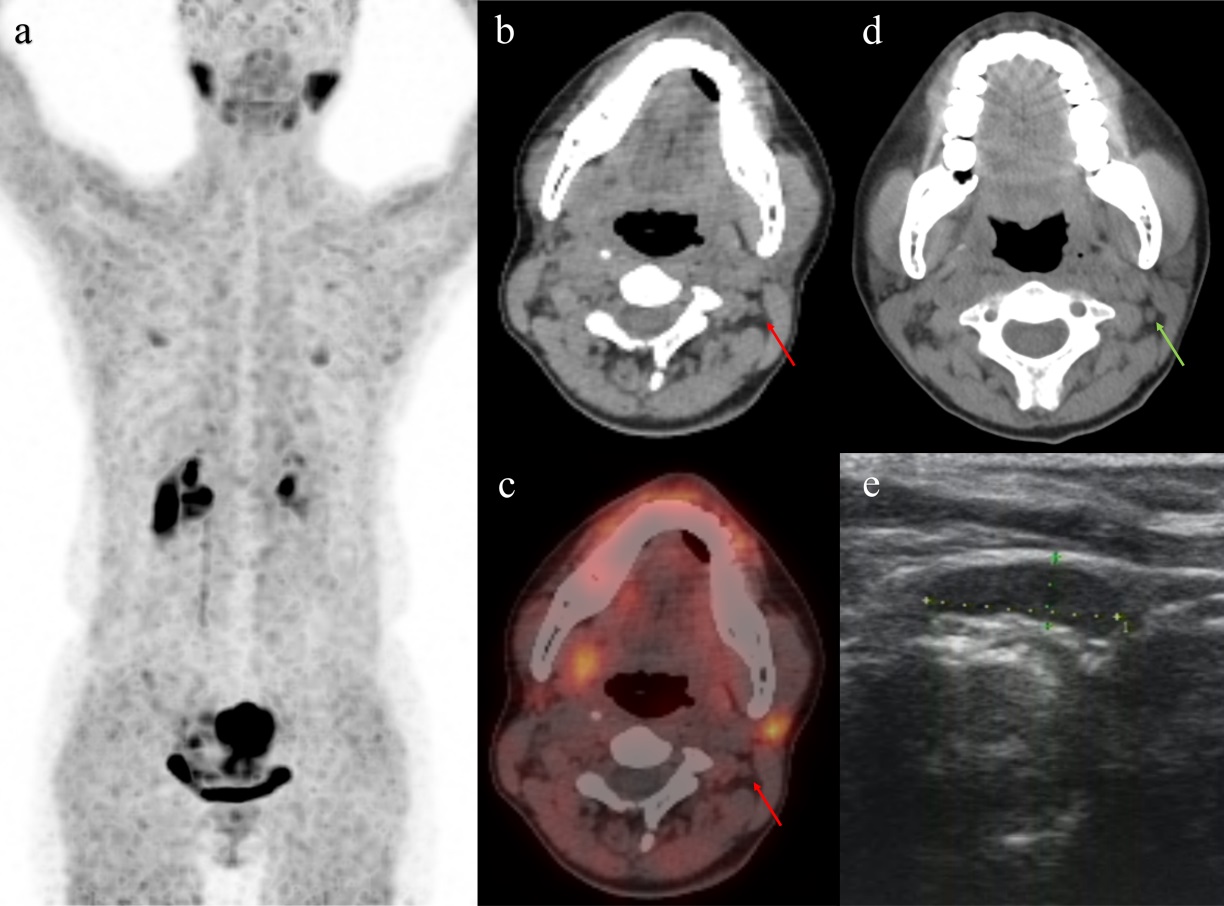  **Supplementary Fig 4.** The representative imaging of a negative patient. (a) The MIP of [^18^F]FAPI-42 PET/CT showed no pathological uptake. (b) The axial CT of [^18^F]FAPI-42 PET/CT exhibited a 5mm short-diameter lymph node in the left neck(red arrow). (c) The fusion imaging of [^18^F]FAPI-42 PET/CT revealed no positive uptake(SUV_max_: 0.8) on this lymph node(red arrow). (d) The lymph node was not depicted morphological change on axial CT of follow-up(green arrow). (e) The follow-up US showed a benign lymph node (5mm short-diameter) in the left neck. | | | | | | | | |
